# Suicide Risk Among US Veterans With Military Service During the Vietnam War

**DOI:** 10.1001/jamanetworkopen.2023.47616

**Published:** 2023-12-28

**Authors:** Tim A. Bullman, Fatema Z. Akhtar, Sybil W. Morley, Julie C. Weitlauf, Yasmin S. Cypel, William J. Culpepper, Aaron I. Schneiderman, Peter C. Britton, Victoria J. Davey

**Affiliations:** 1 Health Outcomes Military Exposures, Veterans Health Administration, Department of Veterans Affairs (VA), Washington, DC; 2 Veterans Integrated Service Network (VISN) 2, Center of Excellence for Suicide Prevention, Department of VA, Washington, DC; 3 VA Palo Alto Health Care System, Menlo Park, California; 4 Stanford University School of Medicine, Stanford, California; 5 Center for Excellence for Suicide Prevention, VA Finger Lakes Healthcare System, Department of VA, Canandaigua, New York; 6 Department of Psychiatry, University of Rochester School of Medicine and Dentistry, Rochester, New York; 7 Office of Research and Development, Veterans Health Administration, Department of VA, Washington, DC

## Abstract

**Importance:**

There are persistent questions about suicide deaths among US veterans who served in the Vietnam War. It has been believed that Vietnam War veterans may be at an increased risk for suicide.

**Objective:**

To determine whether military service in the Vietnam War was associated with an increased risk of suicide, and to enumerate the number of suicides and analyze patterns in suicides among Vietnam War theater veterans compared with the US population.

**Design, Setting, and Participants:**

This cohort study compiled a roster of all Vietnam War–era veterans and Vietnam War theater veterans who served between February 28, 1961, and May 7, 1975. The 2 cohorts included theater veterans, defined as those who were deployed to the Vietnam War, and nontheater veterans, defined as those who served during the Vietnam War era but were not deployed to the Vietnam War. Mortality in these 2 cohorts was monitored from 1979 (beginning of follow-up) through 2019 (end of follow-up). Data analysis was performed between January 2022 and July 2023.

**Main Outcomes and Measures:**

The outcome of interest was death by suicide occurring between January 1, 1979, and December 31, 2019. Suicide mortality was ascertained from the National Death Index. Hazard ratios (HRs) that reflected adjusted associations between suicide risk and theater status were estimated with Cox proportional hazards regression models. Standardized mortality rates (SMRs) were calculated to compare the number of suicides among theater and nontheater veterans with the expected number of suicides among the US population.

**Results:**

This study identified 2 465 343 theater veterans (2 450 025 males [99.4%]; mean [SD] age at year of entry, 33.8 [6.7] years) and 7 122 976 nontheater veterans (6 874 606 males [96.5%]; mean [SD] age at year of entry, 33.3 [8.2] years). There were 22 736 suicides (24.1%) among theater veterans and 71 761 (75.9%) among nontheater veterans. After adjustments for covariates, Vietnam War deployment was not associated with an increased risk of suicide (HR, 0.94; 95% CI, 0.93-0.96). There was no increased risk of suicide among either theater (SMR, 0.97; 95% CI, 0.96-0.99) or nontheater (SMR, 0.97; 95% CI, 0.97-0.98) veterans compared with the US population.

**Conclusions and Relevance:**

This cohort study found no association between Vietnam War–era military service and increased risk of suicide between 1979 and 2019. Nonetheless, the 94 497 suicides among all Vietnam War–era veterans during this period are noteworthy and merit the ongoing attention of health policymakers and mental health professionals.

## Key Points

**Question:**

Is the risk of suicide among US veterans who served in the Vietnam War between 1961 and 1975 greater than that of veterans who served in the military at the same time but not in the Vietnam War theater?

**Findings:**

In this cohort analysis of approximately 9.6 million Vietnam War–era veterans, there was no increased risk of suicide mortality among veterans who served in the Vietnam War theater and veterans who served during the Vietnam War but were not deployed to Vietnam.

**Meaning:**

Findings of this study suggest that although deployment and service during the Vietnam War were not associated with an increased risk of suicide, the high number of suicides among US military personnel between 1979 and 2019 is noteworthy and merits the ongoing attention of health policymakers and mental health professionals.

## Teaser

This cohort study examines the incidence and patterns of suicide from 1979 to 2019 among veterans who were deployed to the Vietnam War and veterans who served in the military at that time but were not deployed to the Vietnam War.

## Introduction

Suicide is a leading cause of death in the US, with close to 50 000 deaths annually.^1,2^ In 2016, the US Department of Veterans Affairs (VA) began publishing national data on veteran suicides in annual suicide prevention reports.^3^ In the 2022 National Veteran Suicide Prevention report, the VA reported that although veterans composed only 7.6% of the US population, they accounted for almost 14% of US suicides.^4^ This report compared sex- and age-adjusted suicide rates in veterans vs nonveterans for each year from 2001 to 2020 and found that suicide rates among veterans remained consistently higher than among nonveterans.^4^ In 2001, the unadjusted annual population-based estimate of suicide mortality was 23.3 per 100 000 persons for veterans vs 12.6 per 100 000 persons for nonveterans. By 2020, the suicide mortality rate had increased to 31.7 per 100 000 persons for veterans vs 16.1 per 100 000 persons for nonveterans.^4^

In the 1980s, media accounts cited figures of more than 50 000 Vietnam War theater veteran suicides in 1981 and 58 000 in 1985.^5,6^ Although these estimates were later found to be incorrect,^7^ questions regarding the association of Vietnam War service with suicide risk emerged and remain. Most published research assessing postservice mortality of Vietnam War theater veterans relied on samples and specific subsets and did not find an association between serving in the Vietnam War and suicide risk.^8,9,10,11,12,13,14,15,16^ Other studies examined theater veteran cohorts with characteristics believed to be associated with greater risk of suicide, including veterans diagnosed with posttraumatic stress disorder^17^ and veterans who were wounded during military service.^18^ Both studies reported an increased risk of suicide among their specific groups of theater veterans. Limitations of the previous literature included a focus on population subsets of veterans from specific military branches^8,9,11^ or veterans who resided in specific states.^12,13,14^ Observation periods in these studies were highly variable.

To address these limitations, this Vietnam era veteran mortality study identified all veterans during the Vietnam War era, between 1961 and 1975, and divided them into 2 cohorts: (1) theater veterans, defined as those who were deployed to the Vietnam War, and (2) nontheater veterans, defined as those who served during the Vietnam War era but were not deployed to the Vietnam War. The suicide risk among these cohorts was evaluated over a 41-year period (1979-2019). The study objectives were to examine (1) baseline statistics on suicide by theater service (ie, theater vs nontheater); (2) crude suicide mortality rates for all theater and nontheater veterans combined, for each theater service group, and for the US male population; (3) adjusted associations between suicide mortality and theater service; and (4) suicide risk among Vietnam War–era veterans stratified by theater service compared with the expected suicide risk for the US general population. The latter analysis differed methodologically from the annual suicide prevention reports by the VA, which compare adjusted suicide rates by calendar year between all veterans and nonveterans.^3,4,19,20^ In contrast, the present study used a cumulative, stratified assessment of suicide risk across calendar years as well as calculated crude rates (CRs) by calendar year among all Vietnam War–era veterans collectively and, when stratified, by theater vs nontheater service. We believe this study makes a unique and comprehensive contribution to existing Vietnam veteran suicide mortality research by examining the universe of Vietnam War–era veterans starting from 1979, with a particular focus on those who served in the Vietnam War theater.

## Methods

The VA Central Institutional Review Board approved this cohort study and waived the informed consent requirement because the study involved minimal risk to study participants. We followed the Strengthening the Reporting of Observational Studies in Epidemiology (STROBE) reporting guideline.

### Vietnam War–Era Cohort Selection

**Figure 1.**
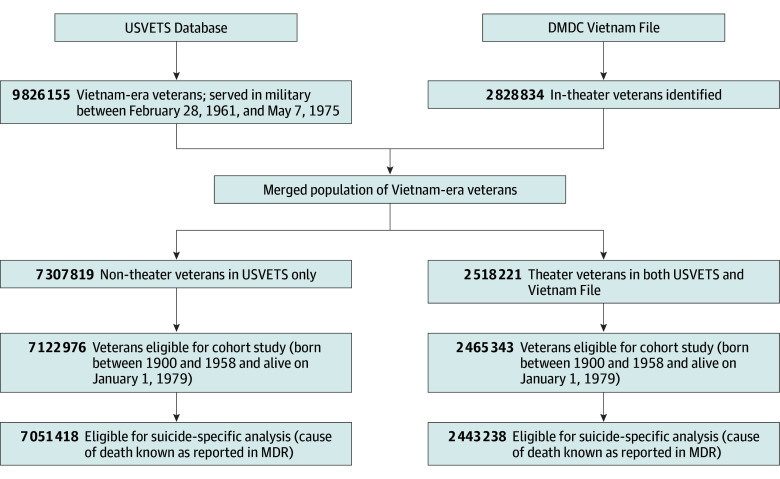
Cohort Selection DMDC indicates Defense Manpower Data Center; USVETS, US Veterans Eligibility Trends and Statistics.

The US Veterans Eligibility Trends and Statistics (USVETS) database^21,22^ was used to identify all veterans (n = 9 826 155) (Figure 1) who served in the US military between February 28, 1961, and May 7, 1975.^23^ Theater service was determined using the Defense Manpower Data Center (DMDC) Vietnam File (n = 2 828 834),^24^ which recorded all veterans who served in Southeast Asia during the Vietnam War. The USVETS database and the Vietnam File included Social Security numbers, demographics, and military service characteristics. Race and ethnicity data were obtained from the USVETS database, which compiled the data from the electronic records of various federal sources, including the US Department of Defense (DOD) and VA. It is not known whether race and ethnicity were self-reported by the veterans at the time of initial contact with the VA or DOD or were based on observation.

Records with a date of birth prior to 1900 or after 1958 and/or a date of death prior to January 1, 1979 (beginning of follow-up for this study), in the USVETS database were excluded. Merging of the records from USVETS database and the Vietnam File resulted in the identification of 2 518 221 theater veterans and 7 307 819 nontheater veterans (Figure 1). After exclusions for birth year and date of death, final counts were 2 465 343 for eligible theater veterans and 7 122 976 for eligible nontheater veterans.

### Vital Status and Cause of Death Ascertainment

The VA/DOD Mortality Data Repository (MDR)^25^ and the USVETS database provided vital status and date of death. The MDR also provided cause of death obtained from annual searches of the National Death Index (NDI) of the National Center for Health Statistics.^26^ At the time of analysis, MDR data were available from 1979 through 2019.

Suicide analyses were limited to records that had been searched in the NDI to enable identification of suicide deaths based on *International Classification of Diseases *(*ICD*) codes. Veterans were excluded if their Social Security numbers were not searched in the NDI. Applying these criteria yielded 2 443 238 eligible theater and 7 051 418 eligible nontheater veterans for inclusion in suicide risk models (Figure 1).

### Statistical Analysis

Follow-up began on January 1, 1979, the earliest date for which cause of death was available, and ended on date of death or December 31, 2019, whichever occurred first. Suicide deaths included *International Classification of Diseases, Ninth Revision *(*ICD-9*) codes E950 to E959 for deaths occurring between 1979 and 1998 and *International Statistical Classification of Diseases and Related Health Problems, Tenth Revision *(*ICD-10*) codes X60 to X84, Y87.0, and U03 for deaths after 1998.^27,28^ The CRs per 100 000 person-years at risk approximated risks of suicide among theater and nontheater veterans separately and all Vietnam War–era veterans collectively.

The outcome of interest for this study was death by suicide occurring between January 1, 1979, the beginning of follow-up, and December 31, 2019, the end of follow-up. Hazard ratios (HRs) and 95% CIs that reflected adjusted associations between suicide risk and theater status were estimated using Cox proportional hazards regression models generated by the PHREG procedure (SAS Institute Inc).^29^ Model covariates were age at entry to follow-up (January 1, 1979), sex (male or female), and race (White or other). Race was based on a composite of race and ethnicity (Hispanic: yes, no, or unknown). White referred to White race and non-Hispanic ethnicity. Other races referred to Hispanic, non-Hispanic Black, and other racial groups (American Indian or Alaska Native, Asian, Hawaiian, and Pacific Islander). White race and unknown Hispanic ethnicity were treated as unknown and excluded from Cox proportional hazards regression models; this coding scheme followed the algorithm used by the Centers for Disease Control and Prevention (CDC) in a study assessing suicide rates by race and ethnicity.^30^

Observations missing 1 or more of the covariates included in the Cox proportional hazards regression models were excluded by SAS from the analysis. Cox proportional hazards regression model analyses were also stratified by branch of service and method-specific suicide risks. The latter included the following suicide methods: (1) intentional poisoning, including intentional drug overdoses (*ICD-9* codes E950-E952; *ICD-10* codes X60-X69); (2) suffocation, including hanging (*ICD-9* code E953; *ICD-10* code X70); (3) firearm (*ICD-9* codes E955.0-E955.4; *ICD-10* codes X72-X74); and (4) all other suicides. The underlying assumption of the Cox proportional hazards regression model (ie, the relative hazard remained constant over time with different variable or covariate levels) was tested both graphically^31^ and statistically^32^ (eAppendix in Supplement 1).

Standardized mortality rates (SMRs) were calculated to compare the observed counts of suicide for all Vietnam War–era veterans collectively and for theater and nontheater veterans separately with the expected counts for the US population (eAppendix in Supplement 1). Specifically, the SMR was the ratio of the number of deaths observed in the veteran cohort to the number of deaths that would be expected if the veteran cohort had the same age-, race and ethnicity–, and sex-specific mortality rates as the US population^33^ (eAppendix in Supplement 1).

The PROC LIFETEST procedure (SAS Institute Inc)^29^ examined differences in crude suicide hazard rates (CRs) for veterans over time by theater service. Hazard rates for both veterans and similarly aged US males, derived from the CDC WONDER database,^34^ were calculated per 100 000 persons at risk (eAppendix in Supplement 1). Changes in temporal patterns of suicide for the veteran cohorts and the similarly aged US male population were further examined using Joinpoint Trend Analysis Software, version 4.0 (National Cancer Institute).^35^ Joinpoint determined the annual percent change (APC) and the average annual percent change (AAPC) in suicide rates since entry into follow-up for veterans and by calendar year for US males based on the best-fit joinpoint regression models selected at an overall α = .05. For veterans, the number of years since entry to follow-up was used as the input for joinpoint and was based on midpoints of calendar years. The initial period of January 1979 to mid-June 1979 (5.5 months) was excluded from the joinpoint analysis because deaths occurring during this period with less than 1 full year of follow-up would not have sufficient follow-up time to support statistical calculations.

Unlike veteran rates, US male suicide rates were enumerated for each calendar year from 1979 to 2019. Because follow-up began on the same date for all veterans (January 1, 1979), calendar year was approximately analogous to the elapsed number of years since entry to follow-up; therefore, the rates for both veterans and male US population were plotted by calendar years.

Two-sided *P* < .05 indicated statistical significance. Data analysis was performed between January 2022 and July 2023, using SAS, version 9.3 (SAS Institute Inc).

## Results

### Demographic and Military Service Characteristics 

A total of 2 465 343 theater veterans and 7 122 976 nontheater veterans were identified. The theater veterans included 2 450 025 males (99.4%) and 15 277 females (0.6%) with a mean (SD) age at year of entry of 33.8 (6.7) years. The nontheater veterans included 6 874 606 males (96.5%) and 245 684 females (3.5%) with a mean (SD) age at year of entry of 33.3 (8.2) years (Table 1). Most Vietnam War–era veterans were identified as non-Hispanic White individuals (78.4% theater vs 76.0% nontheater). Theater veterans compared with their nontheater counterparts were more likely to have been enlisted (90.4% vs 59.7%) and to have served in the Army (56.3% vs 41.5%).

**Table 1.** Demographic and Military Service Characteristics by Theater and Nontheater Status

Characteristic	Veterans, No. (%)	
	Theater (n = 2 465 343)^a^	Nontheater (n = 7 122 976)^a^
Year of birth		
1900-1910	277 (0)	12 995 (0.2)
1911-1920	15 038 (0.6)	130 186 (1.8)
1921-1930	116 668 (4.7)	312 070 (4.4)
1931-1940	347 372 (14.1)	1 038 585 (14.6)
1941-1950	1 768 693 (71.7)	3 619 486 (50.8)
1951-1958	217 101 (8.8)	2 003 270 (28.1)
Unknown	194 (0)	6384 (0.1)
Mean (SD) year of birth	1944.7 (6.6)	1945.2 (8.2)
Mean (SD) age at year of entry (as of January 1, 1979)	33.8 (6.7)	33.3 (8.2)
Sex		
Male	2 450 025 (99.4)	6 874 606 (96.5)
Female	15 277 (0.6)	245 684 (3.5)
Unknown	41 (0)	2686 (0)
Race and ethnicity^b^		
Hispanic	92 795 (3.8)	318 604 (4.5)
non-Hispanic Black	261 729 (10.6)	700 575 (9.8)
non-Hispanic White	1 932 750 (78.4)	5 413 145 (76.0)
Other race and non-Hispanic ethnicity^c^	53 286 (2.2)	135 417 (1.9)
Unknown race and Hispanic ethnicity	30 606 (1.2)	344 251 (4.8)
Known race and unknown Hispanic ethnicity	94 089 (3.8)	209 193 (2.9)
Unknown race and non-Hispanic ethnicity	88 (0)	1791 (0)
Branch of military service		
Army	1 388 214 (56.3)	2 957 728 (41.5)
Marines	346 367 (14.1)	376 560 (5.3)
Air Force	486 450 (19.7)	1 251 967 (17.6)
Navy	241 102 (9.8)	1 586 229 (22.3)
Coast Guard	2902 (0.1)	71 906 (1.0)
Other^d^	7 (0)	9354 (0.1)
Unknown	301 (0)	869 232 (12.2)
Rank		
Enlisted	2 228 986 (90.4)	4 249 372 (59.7)
Warrant officer	13 425 (0.5)	11 490 (0.2)
Officer	221 081 (9.0)	344 156 (4.8)
Unknown	1851 (0.1)	2 517 958 (35.3)

^a^ Some of these veterans were ineligible for suicide analyses but were included in this table to provide a more complete enumeration of the characteristics of all Vietnam War–era veterans. Data on theater veterans were obtained from the US Veterans Eligibility Trends and Statistics database and Defense Manpower Data Center Vietnam File, whereas data on nontheater veterans were from the USVETS database only.

^b^ Race and ethnicity were a composite of administrative data values for the variables of race and Hispanic (yes, no, or unknown) ethnicity. These data were obtained from the US Veterans Eligibility Trends and Statistics database.

^c^ Other races included American Indian or Alaska Native, Asian, Hawaiian, and Pacific Islander.

^d^ Other branches data were from the US Veterans Eligibility Trends and Statistics database only and included the National Oceanic and Atmospheric Administration and US Public Health Service.

Because there were 2 data sources for theater veterans (USVETS database and DMDC Vietnam File) and only 1 source for nontheater veterans (USVETS database), the percentages of missing data were higher among nontheater veterans (Table 1). A substantial portion of theater and nontheater veterans were missing race and ethnicity data (Table 1). Both the Cox proportional hazards regression model and SMR analyses excluded veterans with a missing value for a covariate or a stratifier respectively; thus, the Cox proportional hazards regression model of suicide risk associated with deployment that included race and ethnicity as a covariate excluded 29.8% of theater and 34.2% of nontheater veteran suicides that were missing race and ethnicity (eAppendix in Supplement 1). The SMR analyses also lost observations due to missing race and ethnicity, although the numbers were smaller due to the different coding of race and ethnicity for SMR analysis, which followed the coding scheme for US expected rates. Sensitivity analyses conducted with Cox proportional hazards regression models that allowed for a greater inclusion of observations as a result of differential coding of race and ethnicity produced varied results regarding suicide risk associated with Vietnam War service (eAppendix in Supplement 1). Given the large numbers of both theater and nontheater veterans and the small numbers of veterans missing sex and age, missing sex and age data would have no implications for the reported risk of suicide.

### Overall and Method-Specific Risks of Suicide 

A total of 94 497 suicides were identified, of which 22 736 (24.1%) were among theater veterans (CR, 25.9 per 100 000 person-years at risk) and 71 761 (75.9%) were among nontheater veterans (CR, 28.6 per 100 000 person-years at risk). In adjusted analyses after exclusions for missing covariates, 15 949 theater veterans and 47 176 nontheater veterans were available for Cox proportional hazards regression model analyses (Table 2). For the 41 years between 1979 and 2019, Vietnam War theater status was associated with a decreased risk of suicide mortality among all Vietnam War–era veterans (HR, 0.94; 95% CI, 0.93-0.96) (Table 2). Assessing branch specific suicide risks, only Marines theater veterans did not have a significantly lower risk of suicide (HR, 1.05; 95% CI, 1.00-1.12). Moreover, risk of firearm suicide was not significantly different between theater vs nontheater veterans (HR, 0.99; 95% CI, 0.97-1.01). The leading method of suicide for each of the cohorts based on crude counts (including those excluded from Cox proportional hazards regression models) was by firearm. While graphically, it does not appear that the Cox proportional hazards regression model assumption was violated during the first 35 years of follow-up, statistical analysis did indicate a violation (*P* < .05). However, in a data set as large as this study’s (over 9 million records) and with small correlations (<0.1), statistically significant violations of proportional hazards should be of little practical significance.^36^

**Table 2.** Results of Cox Proportional Hazards Regression Models of Suicide Mortality Risks Associated With Theater Status

Outcome: cohort	No. of veterans^a^	HR (95% CI)
Suicide: all veterans^b^		
Theater	15 949	0.94 (0.93-0.96)
Nontheater	47 176	1 [Reference]
Branch of service		
Suicide: Army only		
Theater	9123	0.86 (0.84-0.88)
Nontheater	20 544	1 [Reference]
Suicide: Marines only		
Theater	2556	1.05 (1.00-1.12)
Nontheater	3281	1 [Reference]
Suicide: Navy only		
Theater	1522	0.84 (0.79-0.88)
Nontheater	11 158	1 [Reference]
Suicide: Air Force Only		
Theater	2732	0.83 (0.79-0.87)
Nontheater	8069	1 [Reference]
Method-specific suicide		
Firearm^c^		
Theater	11 468	0.99 (0.97-1.01)
Nontheater	31 494	1 [Reference]
Suffocation^d^		
Theater	1691	0.86 (0.81-0.91)
Nontheater	6028	1 [Reference]
Intentional poisoning^e^		
Theater	2005	0.88 (0.83-0.92)
Nontheater	7033	1 [Reference]
All other suicide		
Theater	785	0.86 (0.80-0.94)
Nontheater	2621	1 [Reference]

Abbreviation: HR, hazard ratio.

^a^ Excluded veterans with missing data on sex, race and ethnicity, and/or age at entry (ie, date of birth).

^b^ Suicide deaths included those with *International Classification of Diseases, Ninth Revision* codes E950 to E959 and *International Statistical Classification of Diseases and Related Health Problems, Tenth Revision* codes X60 to X84, Y87.0, and U03.

^c^ Firearm suicides included those with *International Classification of Diseases, Ninth Revision* codes E955.0 to E955.4 and *International Statistical Classification of Diseases and Related Health Problems, Tenth Revision* codes X72 to X74.

^d^ Suffocation suicides included those with *International Classification of Diseases, Ninth Revision* code E953 and *International Statistical Classification of Diseases and Related Health Problems, Tenth Revision* code X70.

^e^ Intentional poisoning suicides included those with *International Classification of Diseases, Ninth Revision* codes E950 to E952 and *International Statistical Classification of Diseases and Related Health Problems, Tenth Revision *codes X60 to X69.

### Suicide Mortality Risk Among Veterans vs US Population

None of the veteran cohorts showed increased risk for suicide mortality compared with the US population after adjusting for sex, race and ethnicity, age, and calendar year. There were fewer than expected suicides among the veteran groups separately (theater: SMR, 0.97 [95% CI, 0.96-0.99]; nontheater: SMR, 0.97 [95% CI, 0.97-0.98]) and all Vietnam War–era veterans collectively (SMR, 0.97; 95% CI, 0.97-0.98).

### CRs of Suicide Mortality Over Time 

**Figure 2.**
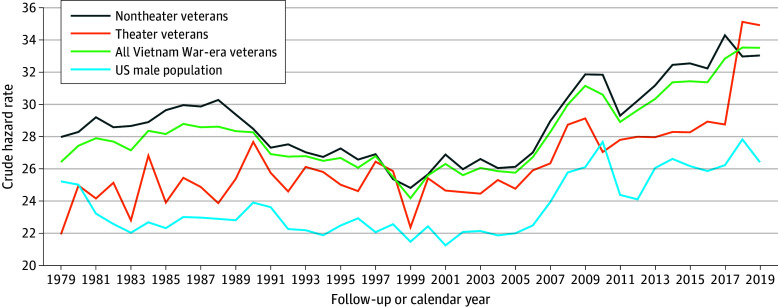
Crude Hazard Rates of Suicide Among Theater, Nontheater, and All Veterans of Vietnam War Era and US Male Population by Number of Years Since Entry to Follow-Up or Calendar Year US male population suicide rates are based on calendar year of death (obtained from CDC Wonder). Veteran suicide rates were calculated for one-year intervals from 1979 to 2019. The first year interval includes 5.5 months to 1.5 years (1 year). All subsequent rates are for time intervals that begin at midpoint and end at midpoint of the subsequent year (eg, 2 years = 1.6 years − 2.5 years). Accordingly, the separate time intervals for veterans, 1 to 41 years, cross calendar years. Crude rates for both US males and veterans are per 100 000 persons.

Figure 2 presents the temporal patterns of suicide rates for Vietnam War–era veterans collectively, for each theater status, and for the US male population by calendar years 1979 to 2019. Deaths during the first period of less than a year were excluded; these 5.5 months included 964 suicides among nontheater veterans and 293 suicides among theater veterans. In 1979, after 1 year of follow-up, the CR of suicide among theater veterans was 21.9 per 100 000 persons, increasing to 34.9 per 100 000 persons in 2019 after 41 years. During the first 38 years of follow-up (1979-2016), there was a significant increase in suicide rates (APC, 0.4; 95% CI, 0.2-0.6), and the AAPC for the entire 41 years also showed a significant increase in rates (AAPC, 1.1; 95% CI, 0.3-1.8).

After 1 year of follow-up, the suicide CR among nontheater veterans was 27.9 per 100 000 persons, increasing to 33.0 per 100 000 persons after 41 years. In comparison with theater veterans, over the entire 41 years of follow-up, there was no overall increase in suicide rates (AAPC, 0.4; 95% CI, –0.2 to 1.1).

Among all Vietnam War–era veterans after 1 year of follow-up, the suicide CR was 26.4 per 100 000 persons, increasing to 33.5 after 41 years of follow-up. There were 3 periods of distinct trends representing an increase in suicide rates: 1979 to 1987 (APC, 0.8; 95% CI, 0.4-1.3), 2005 to 2009 (APC, 4.5; 95% CI, 2.1-7.0), and 2012 to 2019 (APC, 2.1; 95% CI, 1.3-2.8). Over the entire 41 years of follow-up, there was also a significant increasing trend in suicide rates (AAPC, 0.6; 95% CI, 0.1-1.0).

Among US males, the suicide rate in 1979, corresponding to approximately 1 year of follow-up for veterans in this study, was 25.2 per 100 000 persons and increased to 26.4 in 2019, corresponding to 41 years of follow-up for veterans in this study, with no calendar year having a significant change in suicide rates. The suicide rates for veterans and US males exhibited similar patterns of increase and decrease over time as they aged (Figure 2).

## Discussion

To our knowledge, this cohort study is the most comprehensive examination to date of the association between Vietnam War military service and suicide risk. The results showed no association between either Vietnam War–era military service or theater service and increased risk of suicide.

Compared with the expected US population rates using SMRs, suicide risk across 41 years of observation was not increased for theater, nontheater, and all Vietnam War–era veterans collectively. All 3 groups of veterans had a similar deficit of suicides compared with the US population (eAppendix in Supplement 1). In veteran-to-veteran comparisons using Cox proportional hazards regression models, theater veterans had modestly decreased suicide risk vs nontheater veterans, which was robust to adjustment across all branches of military service. There was also no increased risk in method-specific suicide associated with theater status.

Comparing the AAPCs between theater and nontheater veterans, there was no statistical difference between the overall trends between the 2 groups. However, among theater veterans there was a significant increase in AAPC in the 41 years of post-1979 follow-up, and risk increased from age 33 years (mean age at year of entry) through age 71 years at follow-up. Comparing the veteran cohorts to US males revealed that the CRs of suicides among veterans remained higher than the suicide CRs of the US population for each of the 41 calendar years, but the overall trends were similar over time.

Of interest was the finding of no increased suicide risk after 41 years of follow-up among veterans compared with the US population based on the SMR, although higher CRs of suicide by calendar year were also found among the veterans compared with US males. The higher rates by calendar year in veterans are consistent with findings in annual VA suicide reports that compare adjusted suicide rates by calendar year among all veterans to the US population. A recent study^37^ that compared veteran and nonveteran suicide rates using both direct and indirect standardization methods similarly found that estimates of suicide risk among veterans were lower based on indirect standardization (ie, SMR). The CRs by calendar year reported in the present study, the direct adjusted rates by calendar year in the VA suicide prevention reports, and the cumulative estimate of suicide risk (as approximated by the SMR) are all valid estimates of suicide risk. The CRs provided snapshots of rates for each year, whereas the SMR provided cumulative risk estimates wherein the expected number of events was standardized to the veteran cohort (eAppendix in Supplement 1). Furthermore, SMR analyses in this study differed from the annual VA data reports in that they examined rates across 41 years rather than over single years, used indirect vs direct adjustment, and adjusted for race and ethnicity, which were not accounted for in the VA suicide prevention reports. A complete explanation of the methodological differences between these 2 estimates of risk is provided in the eAppendix in Supplement 1.

### Strengths and Limitations

This study has 2 major strengths that distinguish it from prior studies assessing suicide mortality among Vietnam War–era veterans. First, the entire population of Vietnam War–era veterans, rather than samples or subsets, was included. Second, 41 years of follow-up were available, adding 19 additional years to the Vietnam Experience Study on postservice mortality.^9^

The most consequential limitation of the present Vietnam Era veteran mortality study was the absence of mortality data before January 1979. Given that the Vietnam Experience Study,^9^ a 30-year follow-up study of Vietnam War–era veterans, reported the highest suicide rate within the first 5 years after military discharge, the current study may have underestimated the number of suicides among all Vietnam War–era veterans and missed an excess of theater veteran suicides that might have occurred soon after discharge from the military and/or deactivation after the end of deployment. Given the modest decreased risks among theater veterans compared with nontheater veterans (HR, 0.94; 95% CI, 0.93-0.96) and with the US population (SMR, 0.97; 95% CI, 0.96-0.99) as well as the lack of mortality data for years reportedly with the highest suicide rates among Vietnam War theater veterans, any statement of a decreased risk of suicide among theater veterans, although accurate in the context of this study, may not accurately reflect the risk when pre-1979 suicides were included.

Another limitation was the lack of data on potential confounders or exposures (eg, serving in a combat role while deployed). Previous research showed that certain subgroups of theater veterans were at an increased risk for suicide (eg, those diagnosed with posttraumatic stress disorder and those wounded during service).^17,18^ However, the present study does answer the question of whether Vietnam War service in general is associated with suicide risk. Underreporting of suicides on death certificates has been documented.^38,39^ However, underreporting should be random regarding theater and nontheater status.

Results of suicide risk estimates varied by how missing data for race and ethnicity were handled (eAppendix in Supplement 1). Excluding rather than including race and ethnicity (Table 2) from models produced similar suicide risk estimates (ie, no increased risk of suicide associated with deployment). Suicide risks differed primarily by how those with White race or unknown Hispanic ethnicity were identified (ie, missing vs White). Given the large number of observations with missing race and ethnicity and the possibility that missing data may not be random regarding theater vs nontheater status, it was unwise to attempt to impute missing values.

## Conclusions

To our knowledge, this was the first study to include all Vietnam War–era veterans in an assessment of suicide dichotomized by service in the Vietnam War theater. Evaluation of mortality data from 1979 to 2019 revealed no association between theater service and risk of suicide. While individual calendar year suicide rates among veterans were higher than that for the US male population, after 41 years of follow-up, we found that all Vietnam War–era veterans collectively or by theater status did not have increased risks of suicide compared with the US population.

Despite the absence of an increased risk of suicide among theater veterans or among all Vietnam War–era veterans, it is nonetheless sobering to reflect on the 94 497 suicide deaths that have occurred among all Vietnam War–era veterans since 1979. This loss of life deserves not only to be noted on behalf of these veterans and their survivors but also merits the ongoing attention of health policymakers and mental health professionals, especially given that suicide rates have increased over 41 years among all Vietnam War–era veterans, veterans of other eras of military service, and the wider US population.

## Notes

Supplement 1.
eAppendix.

eReference

Supplement 2.
Data Sharing Statement

## References

[1] Centers for Disease Control and Prevention. Preventing suicide. 2022. Accessed July 7, 2023. https://www.cdc.gov/suicide/pdf/NCIPC-Suicide-FactSheet-508_FINAL.pdf

[2] Curtin SC, Hedegaard H, Ahmad FB. Provisional numbers and rates of suicide by month and demographic characteristics: United States, 2020: Vital Statistics Rapid Release, no 16. National Center for Health Statistics. November 2021. Accessed July 12, 2023. https://stacks.cdc.gov/view/cdc/110369

[3] US Department of Veterans Affairs, Office of Mental Health and Suicide Prevention. Suicide among veterans and other Americans 2001-2014. 2016. Accessed July 20, 2023. https://www.mentalhealth.va.gov/docs/2016suicidedatareport.pdf

[4] US Department of Veterans Affairs. 2022 National Veteran Suicide Prevention annual report. Accessed July 12, 2023. https://www.mentalhealth.va.gov/docs/data-sheets/2022/2022-National-Veteran-Suicide-Prevention-Annual-Report-FINAL-508.pdf

[5] Anderson R. Vietnam legacy: veterans’ suicide toll may top war casualties. *Seattle Times*. March 18, 1981.

[6] Langone J. The war that has no ending. Discover. 1985;(June):44-54.

[7] Pollock DA, Rhodes P, Boyle CA, Decoufle P, McGee DL. Estimating the number of suicides among Vietnam veterans. Am J Psychiatry. 1990;147(6):772-776. doi:10.1176/ajp.147.6.772 2343923

[8] Centers for Disease Control Vietnam Experience Study. Postservice mortality among Vietnam veterans. JAMA. 1987;257(6):790-795. doi:10.1001/jama.1987.03390060080028 3027422

[9] Boehmer TK, Flanders WD, McGeehin MA, Boyle C, Barrett DH. Postservice mortality in Vietnam veterans: 30-year follow-up. Arch Intern Med. 2004;164(17):1908-1916. doi:10.1001/archinte.164.17.1908 15451767

[10] Watanabe KK, Kang HK, Thomas TL. Mortality among Vietnam veterans: with methodological considerations. J Occup Med. 1991;33(7):780-785. doi:10.1097/00043764-199107000-00010 1890488

[11] Watanabe KK, Kang HK. Military service in Vietnam and the risk of death from trauma and selected cancers. Ann Epidemiol. 1995;5(5):407-412. doi:10.1016/1047-2797(95)00039-A 8653214

[12] Anderson H, Hanrahan LP, Jensen M, Laurin D, Yick W, Weigman P. Wisconsin Vietnam Veteran Mortality. Wisconsin Division of Health; 1986.

[13] Kogan MD, Clapp RW. Mortality Among Vietnam Veterans in Massachusetts, 1972-1983. Massachusetts Department of Public Health, Division of Health Statistics; 1985.

[14] Lawrence CE, Reilly AA, Quickenton P, Greenwald P, Page WF, Kuntz AJ. Mortality patterns of New York State Vietnam Veterans. Am J Public Health. 1985;75(3):277-279. doi:10.2105/AJPH.75.3.277 3976954 PMC1646175

[15] Forsberg CW, Estrada SA, Baraff A, . Risk factors for suicide in the Vietnam-era twin registry. Suicide Life Threat Behav. 2022;52(4):631-641. doi:10.1111/sltb.12848 35499385

[16] Kang HK, Cypel Y, Kilbourne AM, . HealthViEWS: mortality study of female US Vietnam era veterans, 1965-2010. Am J Epidemiol. 2014;179(6):721-730. doi:10.1093/aje/kwt319 24488510

[17] Bullman TA, Kang HK. Posttraumatic stress disorder and the risk of traumatic deaths among Vietnam veterans. J Nerv Ment Dis. 1994;182(11):604-610. doi:10.1097/00005053-199411000-00002 7964667

[18] Bullman TA, Kang HK. The risk of suicide among wounded Vietnam veterans. Am J Public Health. 1996;86(5):662-667. doi:10.2105/AJPH.86.5.662 8629716 PMC1380473

[19] US Department of Veterans Affairs, Office of Mental Health and Suicide Prevention. 2020 National Veteran Suicide Prevention annual report. Accessed July 20, 2023. https://www.mentalhealth.va.gov/docs/data-sheets/2020/2020-National-Veteran-Suicide-Prevention-Annual-Report-11-2020-508.pdf

[20] US Department of Veterans Affairs, Office of Mental Health and Suicide Prevention. 2021 National Veteran Suicide Prevention annual report. Accessed July 26, 2023. https://www.mentalhealth.va.gov/docs/data-sheets/2021/2021-National-Veteran-Suicide-Prevention-Annual-Report-FINAL-9-8-21.pdf

[21] VHA Data Portal. United States Veterans Eligibility Trends and Statistics (USVETS). Accessed July 19, 2023. http://vhacdwdwhtest12.vha.med.va.gov/DataSources/USVETSData.aspx

[22] VA Information Resource Center (VIReC). United States Veterans Eligibility Trends and Statistics (USVETS): a new data source with socioeconomic variables. 2019. Accessed July 18, 2023. https://www.hsrd.research.va.gov/for_researchers/cyber_seminars/archives/video_archive.cfm?SessionID=3626

[23] Title 10–Armed Forces: this title was enacted by act Aug. 10, 1956, ch. 1041, § 1, 70A Stat. 1. Accessed July 25, 2023. https://www.govinfo.gov/content/pkg/USCODE-2021-title10/pdf/USCODE-2021-title10.pdf

[24] Defense Manpower Data Center. Vietnam File. July 1999. Accessed July 14, 2023. https://aad.archives.gov/aad/content/aad_docs/rg330_dcas_faq_vn.pdf

[25] US Department of Veterans Affairs. Using data to prevent veteran mortality. 2022. Accessed July 10, 2023. https://www.mirecc.va.gov/suicideprevention/documents/VA_DoD-MDR_Flyer.pdf

[26] Hoffmire CABS, Barth SK, Bossarte RM. Reevaluating suicide mortality for veterans with data from the VA-DoD mortality data repository, 2000-2010. Psychiatr Serv. 2020;71(6):612-615. doi:10.1176/appi.ps.201900324 32089080 PMC7489458

[27] World Health Organization. Manual of the International Statistical Classification of Diseases: Inquiries, and Causes of Death, Ninth Revision. World Health Organization; 1977.

[28] World Health Organization. International Statistical Classification of Diseases and Related Health Problems, Tenth Revision. World Health Organization; 1992. 3376487

[29] SAS Institute SAS/STAT. 9.3 Users Guide. SAS Institute Inc; 2011.

[30] Stone DM, Mack KA, Qualters J. Notes from the field: recent changes in suicide rates, by race and ethnicity and age group—United States, 2021. MMWR Morb Mortal Wkly Rep. 2023;72(6):160-162. doi:10.15585/mmwr.mm7206a4 36757870 PMC9925140

[31] Allison P. Survival Analysis Using SAS: A Practical Guide. 2nd ed. SAS Institute Inc; 2010.

[32] Kleinbaum DG, Klein M. Survival Analysis: A Self-Learning Text. 3rd ed. Springer; 2012. doi:10.1007/978-1-4419-6646-9

[33] Breslow NE, Day NE. Statistical methods in cancer research: volume II—the design and analysis of cohort studies. IARC Sci Publ. 1987;(82):1-406. 3329634

[34] Centers for Disease Control and Prevention. CDC WONDER: compressed mortality file. May 12, 2023. Accessed July 21, 2023. https://wonder.cdc.gov/wonder/help/cmf.html

[35] Kim HJ, Fay MP, Feuer EJ, Midthune DN. Permutation tests for joinpoint regression with applications to cancer rates. Stat Med. 2000;19(3):335-351. doi:10.1002/(SICI)1097-0258(20000215)19:3<335::AID-SIM336>3.0.CO;2-Z 10649300

[36] Therneau TM, Grambsch PM. Testing proportional hazards. In: Modeling Survival Data: Extending the Cox Model. Statistics for Biology and Health. Springer; 2000. doi:10.1007/978-1-4757-3294-8_6

[37] Morral AR, Schell TL, Smart R. Comparison of suicide rates among US veteran and nonveteran populations. JAMA Netw Open. 2023;6(7):e2324191. doi:10.1001/jamanetworkopen.2023.24191 37462974 PMC10354669

[38] Bakst SS, Braun T, Zucker I, Amitai Z, Shohat T. The accuracy of suicide statistics: are true suicide deaths misclassified? Soc Psychiatry Psychiatr Epidemiol. 2016;51(1):115-123. doi:10.1007/s00127-015-1119-x 26364837

[39] Snowdon J, Choi NG. Undercounting of suicides: where suicide data lie hidden. Glob Public Health. 2020;15(12):1894-1901. doi:10.1080/17441692.2020.1801789 32744898

