# Errors in the Results and Tables 1 and 2

**DOI:** 10.1001/jamanetworkopen.2024.17524

**Published:** 2024-05-17

In the Original Investigation titled “Suicide Risk Among US Veterans With Military Service During the Vietnam War”^1^ published on December 28, 2023, there were errors in the frequency distribution of theater veterans by branch of military service and the hazard ratios for suicide associated with Vietnam deployment by branch for theater veterans that were reported in the Results section and in Tables 1 and 2. The article has been corrected. The authors have provided an explanation in a Comment.^2^
