# Supplementary material for: Suicide Risk Among US Veterans With Military Service During the Vietnam War
Source: JAMA Netw Open. 2023 Dec 28;6(12):e2347616. doi: 10.1001/jamanetworkopen.2023.47616 (PMC10755619; doi:10.1001/jamanetworkopen.2023.47616)
Supplement: Supplement 1. — eAppendix. eReference [file jamanetwopen-e2347616-s001.pdf]

Supplemental Online Content

Bullman TA, Akhtar FZ, Morley SW, et al. Suicide risk among US veterans with military service during the Vietnam War. *JAMA Netw Open*. 2024;7(1):e2347616. doi:10.1001/jamanetworkopen.2023.47616

eAppendix

eReference

This supplemental material has been provided by the authors to give readers additional information about their work.

© 2024 Bullman TA et al. *JAMA Network Open*

eAppendix

Statistical Analysis

Testing Cox Model Assumption

The underlying assumption of the Cox proportional hazards model, the relative hazard remains constant over time with different predictor or covariate levels, was tested graphically using SAS PROC LIFETEST to plot the log[-log(Survival Probability)]) where the y-axis is log[-log(Survival Probability)] and the x-axis is log(time).[31] It was further assessed statistically using SAS PROC PHREG to calculate and test whether the Schoenfeld residuals are correlated with time or with some function of time, which is one of several prescribed statistical analyses for evaluating the violation of the Cox model assumption.[32]

Source of Expected U.S. Population Rates

Expected U.S. population mortality rates were those available from the National Institute for Occupational Safety and Health (NIOSH). The NIOSH data are based on death certificate data submitted to the National Center for Health Statistics by state vital statistics offices. These data include vital status and cause of death, as well as date of birth, date of death, sex, and race. The expected data for the U.S. population incorporates ICD codes stratified by sex, race, and 5-year calendar time periods from 1960 through the most current year available[1]. The actual calculations of the expected rates are based on indirect standardization, where the rates from the referent group (U.S. population) are stratified by age, sex, and race. These strata specific rates are then multiplied by the number of people in the corresponding strata of the population of interest.

[1] Robinson CF, Schnorr TM, Cassinelli RT, Calvert GM, Steenland NK, Gersic CM, Schubauer-Berigan MK. Tenth revision US mortality rates for use with the NIOSH Life Table Analysis System. Journal of Occupational and Environmental Medicine. 2006 Jul 1:662-7.

SMR Calculation

$$\mathrm{SMR} = \frac{\sum_{i} W_i R_{i1}}{\sum_{i} W_i R_{i0}}$$

Where the variables are defined as follows:
- Ri1 = the stratum-specific rate in the observed cohort (the exposed cohort)
- Ri0 = the stratum-specific rate in the unexposed population
- Wi = stratum-specific person years in the exposed cohort.

© 2024 Bullman TA et al. *JAMA Network Open*

Note that the ratio of observed to expected deaths is equivalent to a ratio of sums of weighted rates, in which the weights for each stratum are the person-years in the exposed group. The numerator is a weighted sum of the observed rates in the exposed population and the denominator is a weighted sum of the rates in the unexposed population.

SMR, 95%, C.I.

We calculated 95% CI for the SMRs using Byar's approximation as follows:

$\mu L=D(1 - 1/9D - Z\alpha/2 /3 D1/2)3$

$\mu U=(D+1) (1 - 1/9(D+1) + Z\alpha/2 /3 (D+1)1/2)3$

where D is the observed number of deaths in the cohort and $Z\alpha/2$ represents the $100(1-\alpha/2)$ percentile of the standard normal distribution.[33]

Crude Rate= (# deaths/pop at risk) X 100,000 population at risk

Hazard rates (Crude rates presented over time) are the number of suicides observed for a specific calendar year divided by the number of all those alive up to and including that calendar year per 100,000 persons.) For veterans hazard rates were calculated by number of years since entry to follow-up (01/01/1979 for all veterans and calendar year (1979-2019) for U.S. males. Given all veterans were followed from 01/01/1979, number of years since entry to follow-up is roughly equivalent to calendar year.

As indicated in the formulas above the primary difference between CRs and SMRs is that CRs uses those alive thru a specific calendar date as a denominator, whereas SMR uses an expected value that is standardized to the cohort of interest.

Results

Missing Data for Other Covariates

Only 4 theater suicides (.02%) and 152 (.2%) non-theater suicides were missing sex. All suicides had age, as this was available from NDI.

Sensitivity Analysis

Among the models tested as part of sensitivity analyses, where the HR is the risk of suicide associated with Vietnam deployment, were: 1) not including race as a covariate (HR=0.90; 95%, C.I., 0.88-0.91), 2) coding White/Hispanic ethnicity unknown as White (HR=1.01; 95%. C.I., 1.00-1.03), and 3) coding missing race/ethnicity as a separate race category (HR=1.08; 95%, C.I., 1.06-1.10).

Discussion

Deficits of Suicide Among Veterans Compared to Expected Based on U.S. Population (SMRs)

The decreased risks of suicide among theater, non-theater, and all Vietnam-era veterans when compared to the expected based on the U.S. population may be related to not having mortality data for the first five years or more after the veteran either left the military or left Vietnam. The decreased risks of suicide might also be due to the “healthy soldier effect”, where health screening to serve in the military and access to medical care either from DOD or VA may create a healthier cohort than the U.S. population.[1]

Comparison of SMRs to Crude Rates Overtime

© 2024 Bullman TA et al. *JAMA Network Open*

As noted, the data indicate that based on the Standardized Mortality Ratio (SMR) analysis, the Vietnam era veterans both collectively and when stratified by in-theater status, are at a decreased risk for suicide compared to the U.S. population. Conversely, the individual calendar year crude rates (CRs) for suicide are consistently higher among the veteran cohorts compared to that for the U.S. population (Figure 2). The latter finding is consistent with that reported by the VA in their annual suicide prevention reports, where adjusted rates of suicide among all veterans are compared to suicide rates for the U.S. population. The findings based on the SMR analyses and the veteran to U.S. population crude suicide rates comparison by calendar year raise the question, Can a cumulative estimate of suicide risk among veterans compared to U.S. population (SMR) show no increased risk of suicide, while the individual calendar year for suicide rates are consistently higher among veterans compared to U.S. population? In fact, both findings can be true. The reason is due to features inherent in the calculation of the different suicide risk estimates.

eReference

1. Kang HK, Bullman TA: Mortality among U.S. veterans of the Persian Gulf War. N Engl J Med 1996, 335: 1498-1504.

© 2024 Bullman TA et al. *JAMA Network Open*
