# Supplementary material for: Suicide Risk Among US Veterans With Military Service During the Vietnam War
Source: JAMA Netw Open. 2023 Dec 28;6(12):e2347616. doi: 10.1001/jamanetworkopen.2023.47616 (PMC10755619; doi:10.1001/jamanetworkopen.2023.47616)
Supplement: Supplement 2. — Data Sharing Statement [file jamanetwopen-e2347616-s002.pdf]

Data Sharing Statement

Bullman. Suicide Risk Among US Veterans With Military Service During the Vietnam War. *JAMA Netw Open*. Published December 28, 2023. doi:10.1001/jamanetworkopen.2023.47616

Data
Data available: No
